# Different Frequency of Cyclic Tensile Strain Relates to Anabolic/Catabolic Conditions Consistent with Immunohistochemical Staining Intensity in Tenocytes

**DOI:** 10.3390/ijms21031082

**Published:** 2020-02-06

**Authors:** Yusuke Kubo, Bernd Hoffmann, Katja Goltz, Uwe Schnakenberg, Holger Jahr, Rudolf Merkel, Gundula Schulze-Tanzil, Thomas Pufe, Mersedeh Tohidnezhad

**Affiliations:** 1 Department of Anatomy and Cell Biology, RWTH Aachen University, Wendlingweg 2, 52074 Aachen, Germanyhjahr@ukaachen.de (H.J.); tpufe@ukaachen.de (T.P.); 2 Institute of Biological Information Processing: IBI-2, Forschungszentrum Jülich, 52425 Jülich, Germany; b.hoffmann@fz-juelich.de (B.H.);; 3 Institute of Materials in Electrical Engineering 1 (IWE1) RWTH Aachen University, 52074 Aachen, Germany; schnakenberg@iwe1.rwth-aachen.de; 4 Department of Anatomy and Cell Biology, Paracelsus Medical Private University, Nuremberg and Salzburg, 90419 Nuremberg, Germany; gundula.schulze@pmu.ac.at

**Keywords:** tenocyte, anabolism, catabolism, mechanical stretch

## Abstract

Tenocytes are mechanosensitive cells intimately adapting their expression profile and hence, their phenotype to their respective mechanomilieu. The immunolocalization and expression intensity of tenogenic, anabolic and catabolic markers in tenocytes in response to in vitro mechanical loading have not been monitored by immunohistochemical staining (IHC). Thus, we investigated the association between IHC intensities, different stimulation frequencies, and tenogenic metabolism using a versatile mechanical stretcher. Primary tenocytes obtained from murine Achilles tendons were transferred to poly(dimethylsiloxane) (PDMS) elastomeric chamber. Chambers were cyclically stretched by 5% in uniaxial direction at a variation of tensile frequency (1 or 2 Hz) for 3 h. After stretching, cell physiology, IHC intensities of tendon-related markers, and protein level of the angiogenesis marker vascular endothelial growth factor (VEGF) were evaluated. Cell proliferation in tenocytes stimulated with 1 Hz stretch was significantly higher than with 2 Hz or without stretch, while 2 Hz stretch induced significantly reduced cell viability and proliferation with microscopically detectable apoptotic cell changes. The amount of scleraxis translocated into the nuclei and tenomodulin immunoreactivity of tenocytes treated with stretch were significantly higher than of non-stretched cells. The collagen type-1 expression level in tenocytes stretched at 1 Hz was significantly higher than in those cultivated with 2 Hz or without stretching, whereas the matrix metalloproteinase (MMP)-1 and MMP-13 immunoreactivities of cells stretched at 2 Hz were significantly higher than in those stimulated with 1 Hz or without stretching. The secreted VEGF-protein level of tenocytes stretched at 2 Hz was significantly higher than without stretching. Our IHC findings consistent with cell physiology suggest that appropriate stretching can reproduce in vitro short-term tenogenic anabolic/catabolic conditions and allow us to identify an anabolic stretching profile.

## 1. Introduction

Tendon acts mechanically as a connection to transmit muscle force to bone [1]. Structural and mechanical integrity of tendons is maintained by changing the metabolic processes in tenocytes [1,2]. Tenocytes generally show an increased synthetic performance under suitable mechanical stimulation. This physiological loading increases an anabolism in tenocytes by collagen production, leading to tenocyte differentiation [3]. Conversely, the microcracks of the tendon induced by repetitive mechanical overloads can accelerate catabolic changes, resulting in tendinopathy [4]. Clinically, the treatment of painful chronic tendinopathy still remains challenging due to the high failure rate of conservative treatment [5,6]. Acute inflammation and mechanical overloads reportedly cause degenerative damages to tendon structure because of qualitative and quantitative tenocyte alterations [7,8]. On the other hand, the angiogenesis-associated signaling pathway plays a key role in tendon remodeling after overloading, which contributes to the pathogenesis of tendinopathy [1,9]. However, the detailed mechanism is controversial. Thus, further elucidation of pathogenetic key events in tendinopathy is expected to improve tendon healing for these patients.

In vitro cyclic mechanical loading on tenocytes is considered to be reasonable to mimic biological environment in tendon tissue because it is one of the regulatory factors affecting tendon physiology. There have been several previous reports investigating the effects of mechanical strain on the gene expression of tenogenic markers. Some studies have shown that cyclic mechanical loading within a physiological range can be beneficial for the tenogenic differentiation and proliferation during the in vitro cultivation of tenocytes [10,11,12,13,14], while others reported that excessive stimulation induced an up-regulation of tenocyte catabolism or apoptosis [15,16,17]. The selection of bioreactor type, amplitude, stimulation time profile, and frequency can affect the extent of stimulation to cells [9,18]. However, the influence of frequency on anabolic/catabolic conditions of tenocytes has been little discussed. Additionally, to the best of our knowledge, there have been no previous in vitro studies to evaluate these conditions focused on immunohistochemical staining (IHC) intensity and distribution.

We hypothesized that (i) optimal stimulation can lead to tenocyte anabolism, whereas high frequency can result in catabolism, and that (ii) IHC intensity for tenocyte-related markers can relate to these cell metabolisms. Therefore, the purpose of our study is to investigate the association between IHC intensities, different stimulation frequencies, and tenogenic metabolism using a versatile mechanical stretcher.

## 2. Results

**Figure 1:**
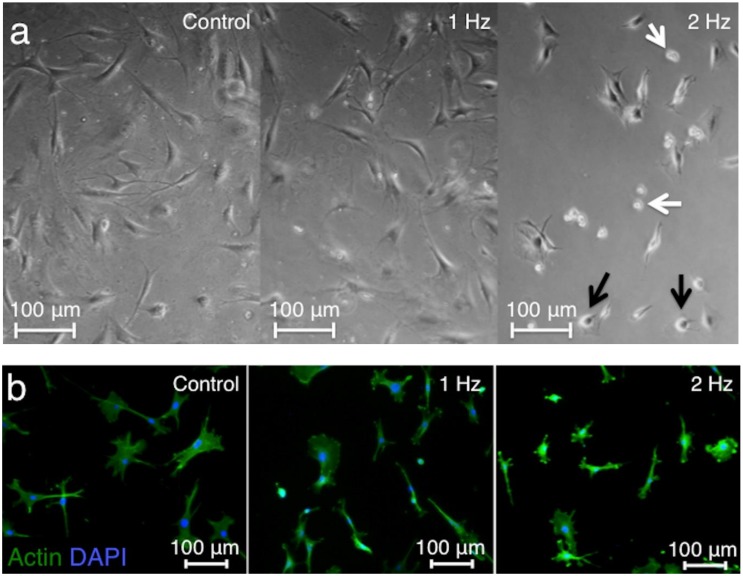
Cell morphology at different tensile frequencies. Cell morphology on light microscopy in the three groups: tenocytes cultured without stretching (control), with stretching at 1 Hz, and with stretching at 2 Hz. A 2 Hz stretch of the cells led to rounded cell shape (black arrows) and floating cells (white arrows) (**a**). Comparison of representative fluorescence stained sections of the three groups depicting F-actin with phalloidin-Alexa 488 (green) (**b**). Cell nuclei were counterstained with DAPI (blue). 5% stretch was applied for 3 h.

The light microscopy showed shortened cell bodies, detached cells, and apoptotic features after stretching at a 2 Hz frequency, whereas elongated cell shape with a central cell nucleus observed in the controls was maintained after stretching at a 1 Hz frequency (Figure 1a). Staining of F-actin by Alexa Fluor 488-labeled Phalloidin was conducted to visualize the cytoskeletal structure of tenocytes. Tenocytes stretched at 2 Hz had small and round shapes, while tenocytes stretched at 1 Hz had similar shape compared to control (Figure 1b).

**Figure 2:**
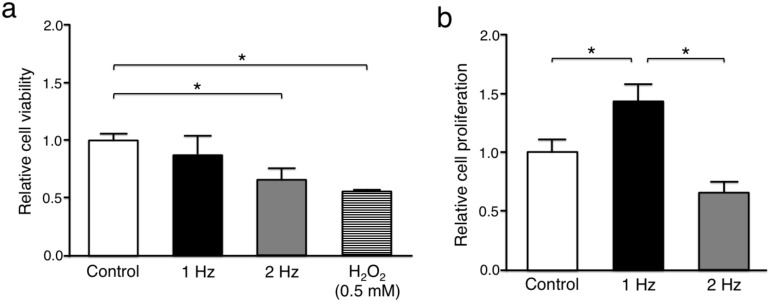
Comparison of cell viability and proliferation at different tensile frequencies. Comparison of cell viability among the three groups: tenocytes cultured without stretching (control), with stretching at 1 Hz, and with stretching at 2 Hz (**a**). 5% stretch was applied for 3 h. The positive control was settled by using 0.5 mM hydrogen peroxide. Comparison of cell proliferation among the three groups (**b**). The value of control was set to 1 (*n* = 5; mean + SEM). * *p* < 0.05 indicates significance. SEM, standard error of the mean; H_2_O_2_, hydrogen peroxide.

The relative cell viability of tenocytes in response to stretching at 2 Hz (0.66 ± 0.10) and hydrogen peroxide (H_2_O_2_) exposure as a positive control (0.53 ± 0.014) was significantly lower than without stretching (1.00 ± 0.059) (*p* < 0.05), whereas there was no significant difference between cyclic stretching at 1 Hz (0.88 ± 0.16) and without stretching (*p* = 0.18) (Figure 2a). The proliferation rate of tenocytes stimulated with a 1 Hz stretch (1.44 ± 0.18) was significantly higher than of those cultured without stretch (1.00 ± 0.12) (*p* < 0.05), whereas tenocytes exposed to stretching at 2 Hz (0.66 ± 0.094) had a significantly lower proliferative activity than those responding to stretching at 1 Hz (*p* < 0.01) (Figure 2b).

**Figure 3:**
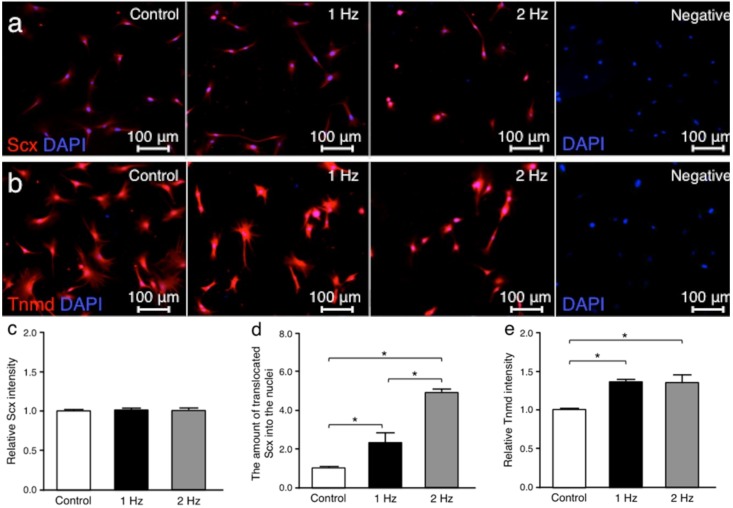
Scleraxis (Scx) and tenomodulin (Tnmd) fluorescence staining in overlay with nucleus coloration at different tensile frequencies. Comparison of representative immunofluorescence-labelings of Scx (red) (**a**) and Tnmd (red) (**b**) among the three groups: tenocytes cultured without stretching (control), with stretching at 1 Hz, and with stretching at 2 Hz. For negative control, primary antibody was omitted. 5% stretch was applied for 3 h. The intensities of Scx (**c**), the amount of translocated Scx into the cell nuclei (**d**), and Tnmd (**e**) were compared among the three groups. Nuclei were counterstained with DAPI (blue). The values of control were set to 1 (*n* = 6; mean + SEM). * *p* < 0.05 indicates significance. SEM, standard error of the mean.

Scleraxis (Scx) and tenomodulin (Tnmd) were investigated since both are tendon-related proteins [18,19]. Immunohistochemically, there was no significant difference in the Scx staining intensities for all conditions between cells cultured without stretching, with stretching at 1 Hz, or with stretching at 2 Hz (without stretching v.s. cyclic stretching at 1 Hz; *p* = 0.34, without stretching vs. stretching at 2 Hz; *p* = 0.11, and stretching at 1 Hz v.s. stretching at 2 Hz; *p* = 0.94, respectively). Conversely, the amount of translocated Scx into the cell nuclei of tenocytes stimulated with a 1 and a 2 Hz stretch was significantly higher than that in cells cultured without stretch (*p* < 0.05 and *p* < 0.01, respectively). The Tnmd intensities in cells exposed to a 1 and a 2 Hz stretch were significantly higher than those maintained without stretch (*p* < 0.01 and *p* < 0.01, respectively) (Figure 3).

**Figure 4:**
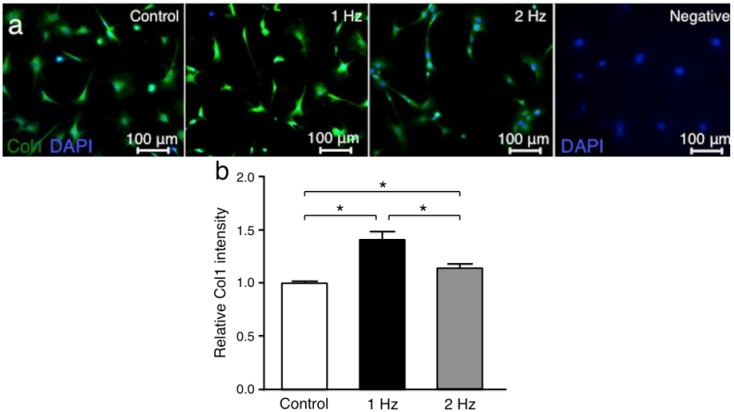
Collagen type 1 (Col1) fluorescence staining in overlay with nucleus coloration at different tensile frequencies. Comparison of representative immunofluorescence labelings of Col1 (green) among the three groups: Cells cultured without stretching (control), with stretching at 1 Hz, and with stretching at 2 Hz (**a**). For negative control, primary antibody was omitted. 5% stretch was applied for 3 h. The intensity of Col1 was compared among the three groups (**b**). Cell nuclei were counterstained with DAPI (blue). The values of control were set to 1 (*n* = 6; mean + SEM). * *p* < 0.05 indicates significance. SEM, standard error of the mean.

Collagen type l (Col1) was investigated since it is the main tendon extracellular matrix protein [20]. The Col1 intensities in tenocytes treated with stretching at 1 Hz and 2 Hz were significantly higher than in those without stretching (*p* < 0.01 and *p* < 0.01, respectively), and the Col1 intensity with stretching at 1 Hz was significantly higher than that with stretching at 2 Hz (*p* < 0.05) (Figure 4).

**Figure 5:**
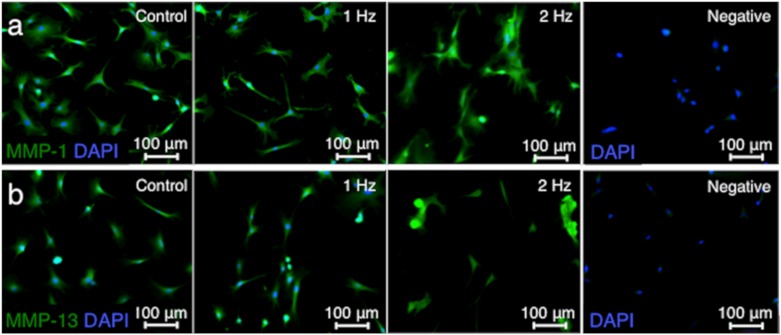

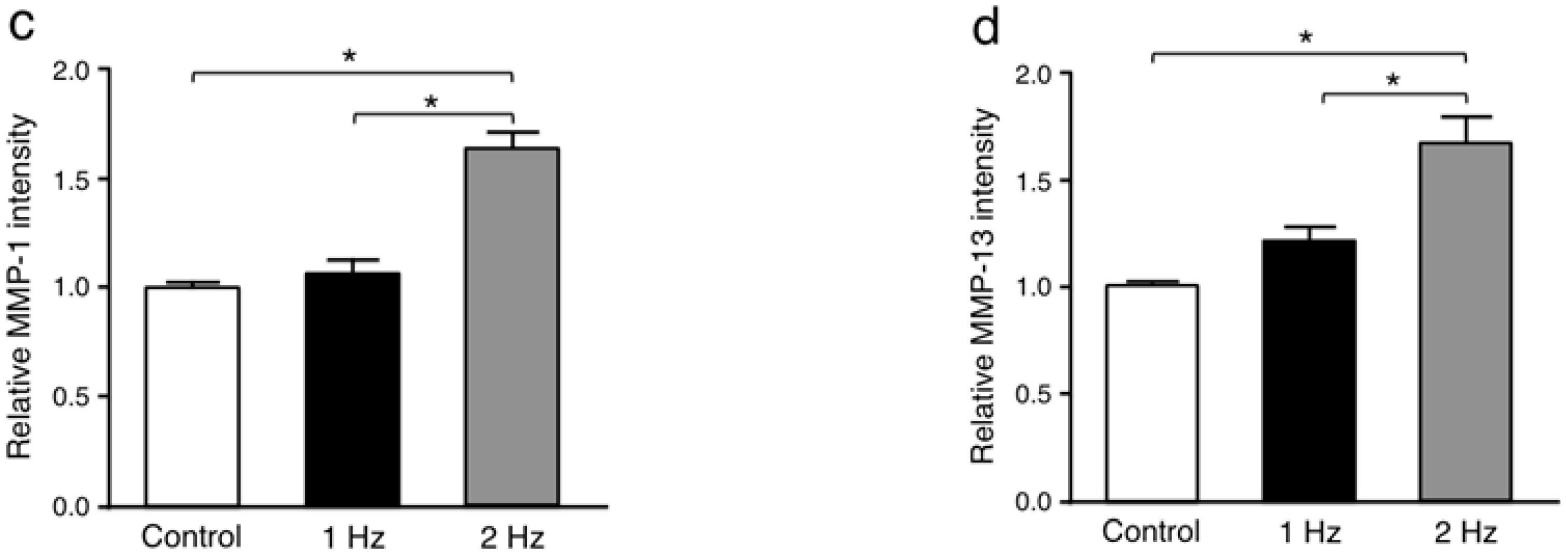
Matrix metalloproteinase (MMP)-1 and MMP-13 fluorescence staining in overlay with nucleus coloration at different tensile frequencies. Comparison of representative immunofluorescence labelings of MMP-1 (green) (**a**) and MMP-13 (green) (**b**) among the three groups: tenocytes cultured without stretching (control), with stretching at 1 Hz, and with stretching at 2 Hz. For negative control, primary antibody was omitted. 5% stretch was applied for 3 h. The intensities of MMP-1 (**c**) and MMP-13 (**d**) were compared among the three groups. Nuclei were counterstained with DAPI (blue). The values of control were set to 1 (*n* = 6; mean + SEM). * *p* < 0.05 indicates significance. SEM, standard error of the mean.

Matrix metalloproteinases (MMP) such as MMP-1 and MMP-13 are responsible for extracellular matrix remodeling by cleaving collagen [21,22]. The MMP-1 and MMP-13 intensities in tenocytes stimulated with a 2 Hz stretch were significantly higher than those cultured without stretch (*p* < 0.01 and *p* < 0.01, respectively) and with a 1 Hz stretch (*p* < 0.01 and *p* < 0.05, respectively), while there was no significant difference in the MMP-1 and MMP-13 staining intensities between cells without stretching and with stretching at 1 Hz (*p* = 0.92 and *p* = 0.06, respectively) (Figure 5).

**Figure 6:**
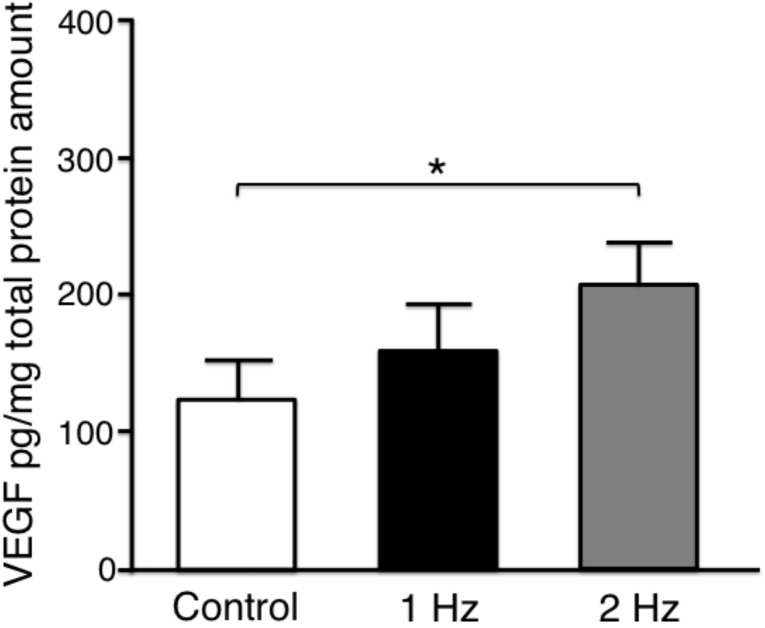
Comparison of the vascular endothelial growth factor (VEGF) concentrations secreted from tenocytes at different tensile frequencies in relation to the total protein. 5% stretch was applied for 3 h. The values were expressed as mean + SEM (pg/mg) (*n* = 7). * *p* < 0.05 indicates significance. SEM, standard error of the mean.

Vascular endothelial growth factor (VEGF) is upregulated during the remodeling process in tendon healing mediating neovascularization [1]. The secreted VEGF-protein level in tenocytes stimulated with a 2 Hz stretch was significantly higher than that without stretch (*p* < 0.05). There was no significant difference between without stretching and with stretching at 1 Hz (*p* = 0.41), and between with stretching at 1 Hz and 2 Hz (*p* = 0.41) (Figure 6).

## 3. Discussion

In our study, 5% (1 mm) mechanical stretch and 1 Hz frequency for 3 h revealed increased cell proliferation. In addition, cell viability and microscopic cell morphology of tenocytes stretched at 1 Hz were similar to those of cells without stretching. Mechanical stimulation parameters such as strain or frequency can strongly affect cellular reactions. Under in vivo and hence, three-dimensional conditions 4–8% strain generally leads to the microscopic tearing of tendon fibers, while macroscopic tendon failure can occur at strain amplitudes above 8% [1,23]. Enhanced gene expression of Col1, decorin, hyaluronic acid, and its receptor (CD44) but no increase in MMP-3 was seen in tenocytes subjected to 5% stretch [24,25,26]. In addition, a 0.5–1 Hz frequency has been adopted in most mechanical studies for tenocytes because these conditions are postulated to be physiological [24,27,28,29,30]. Our mechanical protocol with 5% strain and 1 Hz frequency seemed to be suitable to represent tenocyte anabolism. On the other hand, a 2 Hz frequency was associated with decreased cell viability and proliferation impaired by approximately 60% compared to unstretched cells. Also, shortened cell bodies and some floating cells, an actual drop in viability, were microscopically observed after stretching tenocytes at this frequency. Scott et al. reported that the percentage of apoptotic cells in response to overloading with 20% strain for 6 h using a 1 Hz frequency was significantly higher than that with 1% strain [17]. Therefore, our method of 5% strain and 2 Hz frequency caused excessive stress to cells, resulting in tenocyte catabolic changes. These results suggest that the versatile mechanical stretcher used here can reproduce in vitro short-term tenogenic anabolic as well as catabolic conditions by using different frequencies.

Our IHC data showed significantly higher intensities corresponding with an elevated amount of the transcription factor Scx translocated into cell nuclei and of Tnmd in tenocytes stimulated with 1 and 2 Hz stretch compared to unstretched controls. Scx is a member of the basic helix–loop–helix (bHLH) superfamily of transcription factors, persisting in tenocytes and mesenchymal stem (MSCs) [31]. The protein is considered to be a key protein of tenocyte differentiation and can be detected specifically in differentiated tenocytes within short activation periods reinforced [19]. Scx induces an increased synthesis of Col1 and increases the expression of Tnmd [18]. Tnmd is one of a new family of type II transmembrane glycoproteins, and is used as a further differentiation marker to monitor tenogenic differentiation [18]. Tnmd is associated with increased cell adhesion and fibroblast proliferation [32]. Morita et al. reported that cyclic straining with 5% amplitudes for 48 h induced tenogenic differentiation of human bone marrow-derived MSCs, showing increased Scx expression [12]. On the other hand, Kim et al. reported no significant differences in the Scx and Tnmd expression after 2% and 4% uniaxial stretch for 12 h in tenocytes from rat [33].

In the present study, the Col1 intensity in tenocytes stimulated with a 1 Hz stretch was significantly higher than in those cultured without stretch, while its expression in cells treated with a 2 Hz stretch was significantly decreased compared with that in cells treated with 1 Hz stretch. Col1 is the main component of the tendon as fibrous collagen, and is increasingly formed during tendon formation, fibroblast differentiation, and late tendon pathogenesis [20]. Due to the high proportion of intermolecular cross-linking, matrix quality is improved by the tensile properties of Col1 [34]. The Col1 expression can be initiated by Scx with nuclear factors activating T-cells (NFATc) [35]. Morita et al. reported increased Col1 expression with 10% cyclic strain for 24 and 48 h, whereas the expression was decreased when cells were cyclically stretched by 15% for 48 h [12], which was confirmed by our IHC findings. Therefore, increased intensities of tenocyte differentiation and collagen markers consistent with higher cell proliferation indicate that 5% stretch with a 1 Hz frequency can mimic anabolic conditions in tenocytes.

On the other hand, MMP-1 and MMP-13 intensities in tenocytes treated with a 2 Hz stretch were significantly higher than in those maintained without stretch and exposed to a 1 Hz stretch, while there is no significant difference in the MMP-1 and MMP-13 staining intensities between cells under the conditions without stretching and with stretching at 1 Hz. In the early phase of tendinopathy, inflammation due to the response of the immune system causes damage to the tendon with induction of MMP-1 and MMP-13 expression as tendon catabolic markers [21,22]. Additionally, MMP-1 is one of the collagenases with proteolytic activity in Col1, the most abundant collagen in tendons [36]. Therefore, elevated intensity of MMP-1 in our study may lead to reduced Col1 intensity in tenocytes stretched at 2 Hz. Tsuzaki et al. reported no significant difference in MMP-1 mRNA expression after stretching tenocytes with 3.5% elongation at 1 Hz for 2 h regardless of induced expression of IL-1β, COX2, and MMP-3 [29]. Maeda et al. also reported the down-regulation of MMP-13 expression 24 h after a 2% static strain [13,37]. These previous publications suggest that mechanical stretch within the physiological range (less than 4% strain) can lead to reduced catabolic conditions [1]. In this study, conversely, increased intensities of MMP-1 and MMP-13 were consistent with reduced cell viability and proliferation. These results indicate that exposure of the cells to high 2 Hz frequency at 5% strain can reflect on catabolism in tenocytes.

Our data showed that the VEGF-protein level secreted by tenocytes treated with a 2 Hz stretch was significantly higher than that in cells cultured without stretch, which indicated a similar tendency compared to MMP-1 and MMP-13 intensities. The VEGF expression is accompanied by expression of MMPs [9,38]. Peterson et al. reported that 8% uniaxial stretching at 1 Hz for 24 h increased VEGF expression in rat Achilles tendon fibroblasts, suggesting the involvement of mechanical stress in the regulation of VEGF in tendon tissue [9]. Mousavizadeh et al. also reported that real-time quantitative PCR showed the increase of VEGFA expression within 4 h of cyclic strain (10% strain and 1 Hz frequency) in human tenocytes [28]. Considering that growth factors such as VEGF play an important role in tendon repair processes by enhancing angiogenesis [1], the catabolic conditions after a 2 Hz stimulation frequency in our study may have a potential to proceed the remodeling process by the revascularization through elevated VEGF.

The major limitation of this study was that we examined the cell physiology and IHC intensities of tenocyte-related markers just after stretch. Application of a 2 Hz frequency to tenocytes showed elevated tenocyte-related markers regardless of decreased cell viability by approximately 60%, which indicates the possibility that tenocytes can make remodeling later after being stretched at a 2 Hz frequency. Future study should be undertaken to investigate cell physiology and tenocyte-related marker expression after a later time point after stretch, which would help to elucidate the mechanism of tenocyte remodeling.

## 4. Materials and Methods

### 4.1. Cell Culture

**Figure 7:**
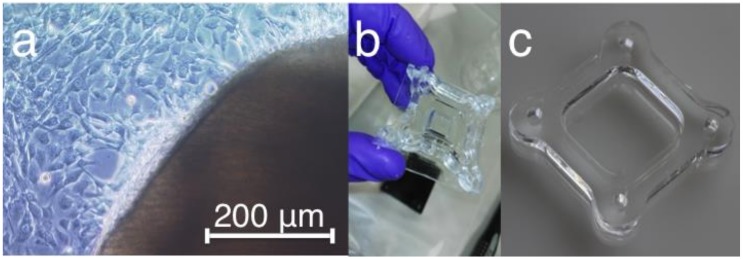
Primary tenocytes and poly(dimethylsiloxane) (PDMS) chamber. (**a**) Cell migration 2 weeks after the Achilles tendon explant cultivation. The light microscopic image of a rat Achilles tendon fragment from which predominantly tenocytes emigrate is shown. (**b**) PDMS elastomeric stretching chambers in mold after polymerization and (**c**) after removal from the mold. The inner area of the stretching chambers has a size of 2 × 2 cm.

The tenocytes used were isolated from Achilles tendons of 6 C57BL/6 mice aged 18−23 weeks. Animal procedures were performed according to German legislation for animal protection (84-02.05.30.13.029, 2017). The preparation was done on ice in phosphate buffered saline (PBS) (2% fungizone and 1% penicillin/streptomycin). The removal of connective tissue cells was carried out with the scalpel on the microscope (DMIL; Leica Microsystems GmbH, Wetzlar, Germany). Subsequently, the Achilles tendon was cut down several times across the longitudinal axis and sterilely transferred into a 50 mL Falcon. The fragments were trypsinized with 1× Trypsin/Ethylenediaminetetraacetic acid (EDTA) for 5 min at 37 °C and centrifuged at 1500 rpm for 5 min at room temperature. After removal of the supernatant, the tendon fragments in 10 mL Dulbecco’s Modified Eagle Medium (DMEM) with 50% fetal bovine serum (FBS), 0.5% fungizone, 1% penicillin/streptomycin were re-suspended and transferred to a petri dish. The cells were incubated at 37 °C in 5% CO_2_ and 97% humidity conditions. Cell culture was controlled according to a gradual reduction of FBS supplementation of 50%, 40%, 30%, 15%, and 10%. Cells were transferred from petri dishes into the T25 or T75 flasks when cell density reached a confluence of about 70−80%. The initially rounded cell bodies at the margin of the explant elongate ellipse-like and migrate to the surface of a cell culture dish 2 weeks after cultivation (Figure 7a).

### 4.2. Poly(dimethylsiloxane) (PDMS) Stretching Chambers

Elastic stretching chambers were made of cross-linked poly(dimethylsiloxane) (PDMS, Sylgard 184, weight ratio 40:1 base to cross-linker) as described before [39] to form a 2.0 × 2.0 cm rectangular frame surrounded by a 5 mm thick wall and consisting a thin (~400 μm) bottom as a chamber for the cells [39]. To realize homogeneity, a degassing step was executed, and subsequently 5 mL of PDMS was carefully poured into each chamber mold. After cross-linked for 16 h at 60 °C, chambers were removed from molds and stored at room temperature. Resulting chambers were linear elastic and characterized by an elasticity of 50 kPa [39,40]. Before use, chambers were coated for 4 h with type-1 collagen (Biochrom GmbH, Berlin, Germany, Cat.No: L7220) in PBS in a ratio of 1:10 on the shaker to facilitate cell adhesion to the elastomeric surface. The sterilization of chambers was performed by exposure to ultraviolet light for 10−20 min (Figure 7b).

For the colonization of PDMS chambers, the cells in the 2nd passage with FBS levels of 10% were used to avoid dedifferentiation of the cells and to guarantee a homogeneous distribution by the trypsinization of the 1st cell passage. 2.5 × 10^4^ cells/cm^2^ were seeded on each PDMS chamber. Subsequently, chambers were filled with 500 μL of DMEM medium with 10% FBS and 1% penicillin/streptomycin.

### 4.3. Mechanical Stretching by Cell Stretcher X6

**Figure 8:**
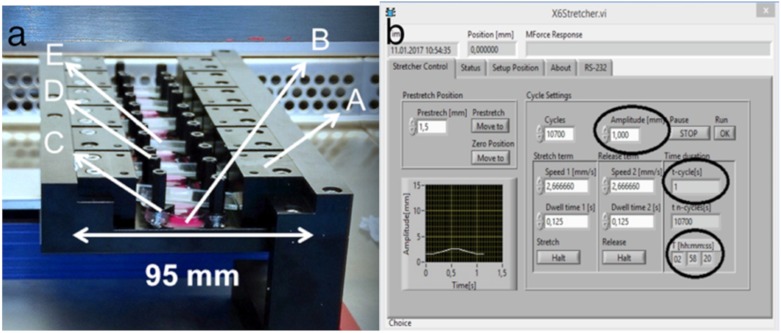
Structure of Cell Stretcher X6 and calibration. Build-up of the X6 stretching device (**a**): A) frame; B) populated silicon chambers with medium; C) Chamber angle; D) Fixing bolts; E) Cover glasses on the left. The manual calibration of the Cell Stretcher X6 with a metal plate on 95 mm as zero position is shown. The calibration was carried out via software (**b**). The control parameters for a 5% (1 mm) amplitude and a 1 Hz frequency are shown as an example (enclosed with a circle). The cycle time (t-cycles) for calculating frequency as an inverse was controlled by the speed 1 and 2. The term of stretching was for 3 h (enclosed with a circle).

After tenocyte adhesion to stretching chambers for 24−36 h, mechanical stretching was conducted with a custom-made 6x device using a linear stepper motor (MT63, Steinmeyer Mechatronik GmbH, Dresden, Germany) (Figure 8a) [39,40]. The mechanical stretcher was calibrated as zero-position to 95 mm. The individual components were disinfected with ethanol and assembled under the clean bench. For the experiments, a maximum of six stretching chambers were clamped, and the same number was carried as controls. Stretching chambers were filled with 500 μL of DMEM medium supplemented with 10% FBS and 1% penicillin/streptomycin. Chambers were fixed on two sides over their entire length. This guaranteed a homogeneous deformation field for the complete analysis area of the chamber. Software settings for amplitudes reached the cells with the same strength as already calibrated before [39,40]. To increase sterility and to reduce evaporation during the experiment, cover glasses were placed on the chambers. The reactor was placed together with control samples in the incubator at 37 °C and wired with the control unit (Figure 8b).

Cyclic stretch experiments were performed with an amplitude of 5% (1 mm) stretch for 3 h. The frequency was varied with 1 Hz and 2 Hz to represent anabolic and catabolic stimulation. Cell morphology after stretching was evaluated by the light microscopy. After completion of the experiment, the PDMS chambers were released from the reactor and used for the corresponding analyses.

### 4.4. Cell Viability Testing

Cell viability after stretching was evaluated by the CellTiter-Blue^®^ Cell Viability Assay (Promega, Madison, WI, USA). The assay is based on the ability of living cells to convert a redox dye (resazurin) into a fluorescent end product (resorufin). By stimulating the final product at 560 nm, more fluorescence is emitted at 590 nm. The measured fluorescence signal, which is approximated to the maxima, is proportional to the number of viable cells. During the stretching test, negative controls were carried out on 12-well plates with different hydrogen peroxide dilutions in medium. The negative controls were relativized by the measurements relative to less viable cells, but it remained in a high-quality form to prevent the cells from detaching. After the stretching test, the culture supernatant was removed, and the reagent in the ratio 1:6 diluted in culture medium was placed in the chambers. Each of the 500 μL detection solution was pipetted and incubated for 90 min. From each sample, 100 μL were pipetted to a 96-well plate as a double determination. Diluted reagent without cells was used as a blank value. Fluorescence signal was detected at 560 nm excitation and 590 nm emission using a fluorescence microplate reader (Infinite M200, TECAN, Salzburg, Austria). Cells were exposed to 0.5 mM of H_2_O_2_ to damage cells as a positive control. After the calculation of the cell amount ratio, the results were normalized to the control group without treatment.

### 4.5. Cell Proliferation Testing

A cell proliferation was performed with the CyQUANT^®^ Cell Proliferation Assay to normalize cell viability after stretching with the actual number of cells per each PDMS chamber. This assay is based on the direct binding of fluorescent dye to the DNA, which is released during a cell lysis. The fluorescence measurement is carried out by the maxima of the excitation at 480 nm and the emission at 520 nm measured using fluorescence microplate reader (Infinite M200, TECAN, Salzburg, Austria). The measured fluorescence is proportional to the number of cells. The implementation was performed according to the protocol of the kit system CyQUANT^®^ Cell Proliferation Assay Kit (Invitrogen, Carlsbad, CA, USA). By means of derogation from the protocol, it was filled to a final volume of 600 μL and pipetted into a 96-well plate as a three-fold determination of 100 μL each. No standard was applied. The measurements were standardized with the initial cell number 2.5 × 10^4^ of the controls. Finally, the results were normalized to the control group without treatment.

### 4.6. Immunohistochemical Staining

The influence of mechanical stretching on tenocyte protein expression was evaluated by the IHC. Tenocyte differentiation was evaluated by using tenocyte-related differentiation markers including Scx and Tnmd [14,15,16]. Additionally, Col1 was used to evaluate collagen production in tenocytes. Conversely, MMP-1 and MMP-13 were assessed as catabolic markers of tenocytes [9,18]. Within immunohistochemical stains, Scx, Tnmd, Col1, MMP-1, and MMP-13 as well as F-actin stress fibers using phalloidin were dyed. Cells on stretched PDMS chambers and unstimulated controls were fixed with 4% formalin solution after washing with PBS. After 6 further washing steps with PBS, the chambers were dried and divided into 4 rectangles with a scalpel. The individual fragments were placed in 12-well plates. They were pre-treated with 0.1% Triton X 100 in PBS to make intracellular protein binding sites more accessible for the antibodies. After 3 washing steps with Tris buffered saline (TBS) for 5 min each, each fragment was blocked for 1 h with 1.5% bovine serum albumin (BSA) in TBS. Non-specific binding points were blocked in order to specify the antibody antigen binding better. Subsequently, primary antibodies against Scx (Santa Cruz Biotechnology, Heidelberg, Germany: sc-87425), Tnmd (Santa Cruz Biotechnology, Heidelberg, Germany: sc-49325), Col1 (Calbiochem, San Diego, CA, USA; 234167), MMP-1 (Abbiotec, San Diego, CA, USA: 250750), and MMP-13 (Abbiotec, San Diego, CA, USA: 251219) were added directed against the respective markers and were diluted in a ratio of 1:200 for Scx, Tnmd, and Col1, 1:350 for MMP-1, and 1:400 for MMP-13 in blocking solution. After an incubation overnight at 4 °C, the coloration was continued with 3 washing steps with TBS for 5 min each. After that, the secondary antibodies Alexa Fluor 488 from donkey against rabbit (Invitrogen, Carlsbad, CA, USA; A21206) for Col1, MMP-1, and MMP-13, and Alexa Fluor 555 from rabbit against goat (Invitrogen, Carlsbad, CA, USA; A21431) for Scx and Tnmd were diluted in a ratio of 1:350 in blocking solution. To recognize possible false positive reaction, a negative control in each staining was performed. For negative control, primary antibody was omitted. The secondary antibody was incubated for 1 h on the shaker. To visualize the organization of actin stress fibers, Alexa Fluor 488 (Invitrogen, Carlsbad, CA, USA; A12379)-labeled Phalloidin was diluted in a ratio of 1:50 in blocking solution. After a further 3 washing steps with TBS for 5 min each, a nuclear coloration with bisbenzimide in PBS was performed in a dilution of 1:200 for 10 min in the individual discolorations. This was followed by 2 washing steps with distilled water for 5 min. Finally, the fragments were capped with Immu-Mount (Thermo Scientific™, Waltham, MA, USA: 9990402).

The evaluation of the IHC was twice carried out microscopically. Images were made using a Keyence BZ-9000 microscope with a 10x or 20x PlanFlour El NA 0.45 Ph1 objective and equal exposure time. In addition, the analytical spectrum was expanded by co-coloring of several proteins. The intensity of staining was expressed as a mean value of three different regions (center, middle-peripheral region, and peripheral region) of the chamber. Fluorescence intensity was evaluated by the ImageJ software (National Institutes of Health, Bethesda, MD, USA) based on the previous report [41]. The mean grey value was determined per cell and the background mean was subtracted. Afterwards, a mean of all cells per image was calculated. A total of 6 images (2 images per region) were used per group and experiment. The fluorescence intensity is a linear grade and can be used for determination of protein levels when the same exposure settings are used for acquisition [42]. Finally, the values were normalized to the control group without treatment. Additionally, the amount of translocated Scx into the cell nuclei was analyzed. We counted the whole DAPI positive nuclei, the Scx positive cells, and Scx-overlapped nuclei (violet nuclei) in three different regions of the chamber.

### 4.7. VEGF Total Protein Determination by ELISA

The level of VEGF released by tenocytes after mechanical stretching was evaluated using ELISA. To analyze the release of VEGF in the supernatant, cells were washed after treatment and cultivated for another 24 h with serum-starved medium based on the previous report [9]. The supernatant was collected and centrifuged for 1 min at 18,000 g to remove the cell debris. Aliquots of the conditioned medium were assayed for VEGF content. The total protein concentrations were determined using bicinchoninic acid (BCA) Kit (Thermo Scientific™, Waltham, MA, USA). The same protein concentrations were used, and the VEGF levels were analyzed by mouse VEGF DuoSet ELISA DY 493-05 (R&D Systems, Minneapolis, MN, USA) according to manufacturer’s instructions.

### 4.8. Statistical Analysis

Cell viability, cell proliferation, intensity of IHC, and VEGF protein amount were compared among three groups including control (tenocytes cultured without stretching), tenocytes stretched with 1 Hz frequency, and stretched with 1 Hz frequency using the nonparametric Wilcoxon rank sum test. Values were expressed as means + standard error of the mean (SEM). Statistical significance was set at *p* < 0.05. All statistical analyses were performed using the GraphPad Prism 6.0 software (La Jolla, CA, USA).

## 5. Conclusions

A short-term uniaxial stretching of tenocytes enhanced cell proliferation with increased Scx and Tnmd intensities in response to a 1 Hz stimulation frequency, whereas reduced cell viability and proliferation with increased MMP-1 and MMP-13 intensities occurred after applying a 2 Hz frequency. Our IHC findings consistent with cell physiology suggest that a novel mechanical stretcher used in this study can have the potential to reproduce in vitro short-term tenogenic anabolic/catabolic conditions by applying the stretch to tenocytes with different frequencies. Therefore, our device would be useful for further in vitro studies to make clear the pathological mechanism of tendinopathy.
